# Correlation between Elemental Composition/Mobility and Skin Cell Proliferation of Fibrous Nanoclay/Spring Water Hydrogels

**DOI:** 10.3390/pharmaceutics12090891

**Published:** 2020-09-18

**Authors:** Fátima García-Villén, Rita Sánchez-Espejo, Ana Borrego-Sánchez, Pilar Cerezo, Lucia Cucca, Giuseppina Sandri, César Viseras

**Affiliations:** 1 Department of Pharmacy and Pharmaceutical Technology, Faculty of Pharmacy, University of Granada, Campus of Cartuja, 18071 Granada, Spain; fgarvillen@ugr.es (F.G.-V.); mcerezo@ugr.es (P.C.); 2 Andalusian Institute of Earth Sciences, CSIC-UGR (Consejo Superior de Investigaciones Científicas-Universidad de Granada), Avenida de las Palmeras 4, Armilla, 18100 Granada, Spain; ritase@correo.ugr.es (R.S.-E.); anaborrego@iact.ugr-csic.es (A.B.-S.); 3 Department of Chemistry, University of Pavia, viale Taramelli 12, 27100 Pavia, Italy; lcucca@unipv.it; 4 Department of Pharmaceutical Sciences, Faculty of Pharmacy, University of Pavia, viale Taramelli 12, 27100 Pavia, Italy; g.sandri@unipv.it

**Keywords:** sepiolite, palygorksite, spring water, hydrogel, wound healing, proliferation, Franz cell, bioactive elements

## Abstract

Inorganic hydrogels formulated with spring waters and clay minerals are used to treat musculoskeletal disorders and skin affections. Their underlying mechanism of action for skin disorders is not clear, although it is usually ascribed to the chemical composition of the formulation. The aim of this study was to assess the composition and in vitro release of elements with potential wound healing effects from hydrogels prepared with two nanoclays and natural spring water. In vitro Franz cell studies were used and the element concentration was measured by inductively coupled plasma techniques. Biocompatibility studies were used to evaluate the potential toxicity of the formulation against fibroblasts. The studied hydrogels released elements with known therapeutic interest in wound healing. The released ratios of some elements, such as Mg:Ca or Zn:Ca, played a significant role in the final therapeutic activity of the formulation. In particular, the proliferative activity of fibroblasts was ascribed to the release of Mn and the Zn:Ca ratio. Moreover, the importance of formulative studies is highlighted, since it is the optimal combination of the correct ingredients that makes a formulation effective.

## 1. Introduction

Inorganic hydrogels formulated with spring waters and nanoclays are successfully used in the treatment of musculoskeletal disorders and skin affections. There is a general agreement that their therapeutic activity against musculoskeletal disorders is achieved through physical mechanisms such as thermic activity, osmotic pressure and electric conductivity [1,2,3,4,5]. On the other hand, the underlying mechanism of action responsible for the therapeutic skin effects are usually ascribed to the chemical composition of the formulation [1,6,7,8,9,10], although the exact therapeutic activities and mechanisms of action are still unknown.

Several dermatological affections have been successfully treated by formulations that include clay minerals [4,11,12,13,14]. Currently, special attention is being paid to wound healing treatments, in which clay minerals have been demonstrated to be very useful [15,16,17,18]. During administration of the formulation, elements from the hydrogel could permeate and/or penetrate across the skin barrier. In a previous study, hydrogels prepared with two different fibrous nanoclays were shown to be fully biocompatible and to exert in vitro wound healing activity [17]. More particularly, it was demonstrated that the fibrous nanoclay hydrogels promoted in vitro fibroblast mobility during wound healing processes.

It is well known that adequate concentrations of certain elements, including Ca, Mg, Na and K, in the wound bed are important for enhancing the healing process [19,20,21,22,23,24,25,26,27]. Transition metals such as Cu, Zn, Mn, Fe, Ag, and Au (among others) have also been demonstrated to play different biological functions in tissue regeneration, as reviewed by Yang et al. [28]. It has also been demonstrated that Zn:Ca ratios reach their maximum during the proliferative stage of wound healing and then decline during the remodeling stage [21]. Moreover, manganese-rich spring waters have been demonstrated to possess wound healing activity [29], and changes in Mg:Ca ratios are essential for a proper wound healing cascade. Consequently, formulations providing adequate bioavailability of elements with wound healing activity will promote the healing process and speed up restoration of the damaged area.

Based on these premises, the aim of this study was to assess the in vitro release and mobility of elements with potential wound healing effects from hydrogels formulated with spring waters and nanoclays that have recently been demonstrated to enhance fibroblast mobility [17]. In vitro Franz cell studies were performed in order to reproduce the topical administration of the formulations and elemental concentration was measured by inductively coupled plasma techniques. The results will be discussed on the basis of both the legal status of elements present in the formulation and their potential therapeutic effects.

## 2. Materials and Methods

### 2.1. Materials

Nanoclay/spring water hydrogels were prepared by mixing Alicún thermal station spring water (ALI, Granada, Spain) with two commercial fibrous nanoclays; sepiolite (PS9) and palygorskite (G30). Nanoclays were kindly gifted by the TOLSA group (Madrid, Spain).

Sepiolite hydrogel included in this study was prepared with a concentration of 10% (*w*/*w*) of PS9 dispersed in ALI spring water (ALIPS9, 250 g in total). Additionally, two palygorskite hydrogels (250 g each), ALIG30@10 and ALIG30@20, were also obtained and their final concentration was 10% *w*/*w* and 20% *w*/*w* of G30, respectively. The three formulations were prepared by means of a turbine high-speed agitator (Silverson LT, Chesham, UK) equipped with a high-traction stirrer head of square mesh and working at 8000 rpm for 5 min.

### 2.2. Methods

#### 2.2.1. Elemental Characterization of Pristine Materials

The elemental composition of ALI, PS9 and G30 was obtained by two Inductively Coupled Plasma techniques: ICP-OES (Optima 8300 ICP–OES Spectrometer, Perkin Elmer, Waltham, MA, USA) and ICP-MS (NexION-300d ICP mass spectrometer, Perkin Elmer), equipped with a triple cone interface and a quadrupole ion deflector using argon for plasma formation. PS9 and G30 were subjected to acid digestion in strong acids (HNO_3_ and HF at a 3:5 ratio, Sigma-Aldrich, MO, USA) inside a Teflon reactor, placed in a microwave oven (Millestone ETHOS ONE, Sorisole, Italy). Calibration curves for ICP-OES were obtained by means of standards solution of 1000 ppm for each element. For ICP-MS, single-element standard solutions (Merck, Darmstadt, Germany) were prepared after dilution with 10% HNO_3_. Ultrapurified water (milliQ grade) was used in both techniques.

#### 2.2.2. In Vitro Release of Elements

Element mobility from ALIPS9, ALIG30@10 and ALIG30@20 was studied by in vitro release studies performed in Franz diffusion cells system (FDC40020FF, BioScientific Inc., Phoenix, AZ, USA) [30]. This system is purposely designed to reproduce dermal and/or mucosal administration conditions. The Franz diffusion cells possessed a contact area of 0.64 cm^2^ and a total volume of 6.4 mL. Dialysis membranes (cut-off 12–14 kDa, 31.7 mm, Medicall International, London) were used to separate the donor and receptor chambers. The membranes were boiled in ultra-purified water (milli-Q water, ISO 3696) for 10 min in order to hydrate them. Over the membrane, in the donator chamber, known amounts of each hydrogel (approximately 0.025 g) were placed. The receptor chamber was filled with degassed, ultra-purified water. The whole system was maintained at a constant temperature of 32 ± 0.5 °C through thermostatic bath circulation. The experiment lasted for 30 min, which is the typical time of topical nanoclay/spring water hydrogels application. Experiments were performed in sextuplicate. At the end of the experiments, the aqueous content of the receptor chamber was carefully withdrawn and filtered through 0.45 μm single-use, syringe filters (Merck Millipore, Madrid, Spain). Finally, the elemental composition on each sample was assessed by ICP-OES. Element release tests were performed after 48 h and 1 month after hydrogel preparation, in order to study the evolution of the elemental mobility. Hydrogel batches were preserved in static conditions inside closed polyethylene containers, which were placed inside a drawer with an average mean temperature of 20 ± 5 °C. Blanks were also analyzed in order to monitor the elements coming from the materials and the ultra-purified water.

#### 2.2.3. Biocompatibility of ALIG30@20

ALIPS9 and ALIG30@10 hydrogels (both with a solid concentration of 10%) have been demonstrated to be biocompatible against fibroblasts [17]. Moreover, in the very same study, the in vitro scratch assay proved that the hydrogels were able to accelerate wound closure by favoring fibroblast migration. Nonetheless, the ALIG30@10 hydrogel showed insufficient viscosity, as proven in another study that included a full rheological characterization of ALIPS9 and ALIG30@10 hydrogels [30]. The low consistency of a hydrogel could hinder its topical administration due to excessive fluidity of the formulation. Consequently, the ALIG30@20 hydrogel was prepared and its biocompatibility was evaluated. To do so, the methodology described by García-Villén et al. [17] was used. Normal human dermal fibroblasts (NHDFs, PromoCell GmbH, Heidelberg, Germany) were seeded and cultured in Dulbecco’s modified Eagle medium (DMEM, Sigma Aldrich^®^-Merck, Milan, Italy), supplemented with 10% fetal bovine serum (FBS, Euroclone, Milan, Italy), 200 IU/mL penicillin and 0.2 mg/mL streptomycin (PBI International, I). Once cellular confluence was obtained (area 0.34 cm^2^/well, density 10^5^ cells/cm^2^), ALIG30@20 was added to the cell culture in concentrations ranging from 1000 to 5 μg/mL and kept in contact with cells for 24 h. Then, the MTT test (3-(4,5-dimethylthiazol-2-yl)-2,5-diphenyltetrazolium bromide) was performed. DMEM phenol red-free and 50 µL of MTT dissolution were added in each well, the final MTT concentration being 2.5 mg/mL. MTT-NHDF contact was maintained for 3 h before the whole supernatant was withdrawn and substituted by 100 µL of dimethyl sulfoxide solution (DMSO, Sigma-Aldrich^®^-Merck, Milan, Italy) to dissolve formazan. The absorbance of each well was measured at 570 nm with an ELISA plate reader (Imark Absorbance Reader, Bio-rad, Hercules, CA, USA), with the reference wavelength set at 655 nm. Fibroblast viability was calculated with respect to the viability of the corresponding control (fibroblasts cultured in fresh DMEM, abbreviated as GM). MTT tests over ALIPS9, ALIG30@10 and ALIG30@20 were performed after 1 month of hydrogel preparation.

#### 2.2.4. Selection of Elements Under Study

**Figure 1:**
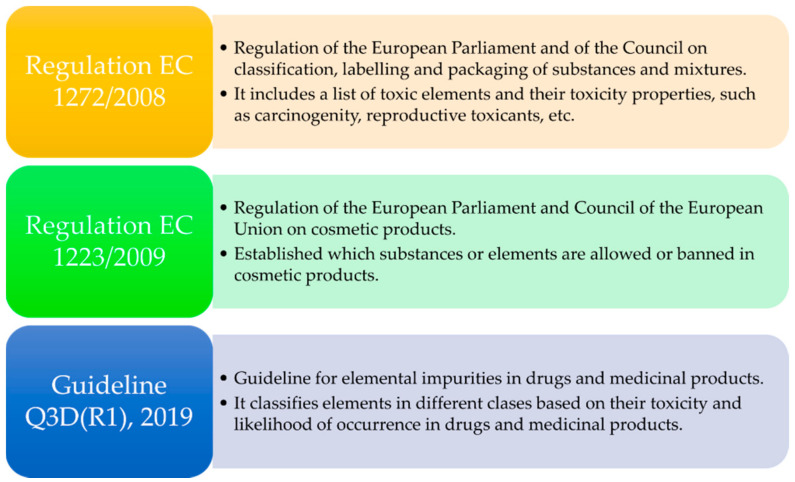
Main regulations [31,32,33] and guidelines used for the selection of elements, interpretation and discussions of results, ordered by year of publication or latest update.

A wide variety of elements were analyzed in this study. In order to organize and facilitate the interpretation of the results, the discussion will be centered around two main aspects: the potential wound healing activity of the elements and their legal situation regarding cosmetics and medicines regulations. The importance of the latter point lies in the fact that, depending on the final therapeutic activity of the present hydrogels, they could be considered as cosmetics or as medicines [31]. Elements will be classified and addressed according to the European regulations and guidelines summarized in Figure 1. The present study is focused on those elements that are considered “safe” or “non-hazardous”. Additionally, elements without toxicity limits (most of the time not mentioned in the aforementioned regulations) were also included in this study.

The guideline for elemental impurities Q3D(R1) [32] of the European Medicines Agency is focused on toxic elements and classifies them in three groups. In view of their limitations and toxicity, all of them with well-defined “permitted daily exposure” (PDE) limits, these elements are not addressed in this manuscript. Nonetheless, there is also a non-defined fourth group that includes elements with low inherent toxicity, without PDE limits. In conclusion, elements in this group should be controlled more for the quality of the final product than for high toxicity and safety considerations. Examples of these elements are Al, B, Ca, Fe, K, Mg, Mn, Na, W and Zn, which are the subject of study of this research. For simplicity throughout the manuscript, these elements are referred to as “class 4”. The European Regulation EC 1223/2009 [33] was used to determine those elements whose presence is either allowed or not mentioned in cosmetic products. 

#### 2.2.5. Statistical Analysis

Statistical analysis were determined by means of non-parametric Mann–Whitney (Wilcoxon) W test. In all cases, SPSS Statistic software (IBM, version 21, 2012, New York, NY, USA) was used and differences were considered significant at *p*-values ≤ 0.05.

## 3. Results and Discussion

### 3.1. Elemental Characterisation of Pristine Materials

Elemental composition of pristine components (PS9, G30 and ALI) is reported in Table 1. According to the EC 1272/2008, any of the detected elements are not considered as carcinogens.

Major elements in the pristine water (ALI) were Sr, S, Ca, Mg and Na (from higher to lower concentrations). The high presence of S, Ca and Mg are in agreement with the nature of the spring water source [34,35]. Ti, Mn, Mg, Sr, Zn and Al are the major elements present in PS9 and G30. In particular, Zn, Mn, Mg and Al belong to class 4 in the Q3D(R1) guideline [32]. Regarding the cosmetic regulation EC 1223/2009 [33], aluminum is the only one specifically allowed in cosmetics, the rest of them are not mentioned in this regulation. Cu and Ag are elements present in the pristine ingredients that have a “special situation” as far as regulation is concerned, since their presence is allowed in cosmetics (mainly due to their role as colorants) but they are classified as class 3 and 2B by the Q3D(R1).

### 3.2. In Vitro Release of Elements

Elements released from ALIPS9, ALIG30@10 and ALIG30@20 hydrogels are summarized in Table 2. As expected from the nature and composition of the pristine ingredients of both hydrogels, the release of major elements (Ca, K, S, Mg, Na) was not only confirmed but desirable due to their physiologic activities, which will be discussed later. In particular, Ca showed significant release levels in all hydrogels, which is in agreement with the high levels of this element in pristine materials (Table 1). Moreover, S is the major element present in ALI, which also explains the high release levels of this element from the formulations.

Release levels of Mg were very similar for the three hydrogels. The release of Al increased with time in all cases, not being detected in any of the young hydrogels. On the other hand, the amount of B released after 1 month was lower. The most remarkable release regarding trace elements was shown by Zn and Sr, followed by Cu. Cu release significantly decreased after 1 month in the three hydrogels. As previously reported, the amount of Cu detected in G30 was higher than PS9 (Table 2). This was in agreement with the lower release of both elements in ALIPS9 versus ALIG30@10 and ALIG30@20. Mn release increased with time in ALIPS9 and ALIPS9@20, while it was under the detection limit of the technique for ALIG30@10 experiments. Levels of Mn were the same for both PS9 and G30 (and absent in ALI, Table 1) but ALIG30@20 showed a remarkably higher release of this element.

The rest of the elements were not released or released in very low amounts. Except for Au, Cu and Ag, the rest of the trace elements are not included/mentioned in the EC 1223/2009 regulation [33]. This means that their safety has not been thoroughly assessed or their toxicity is considered non-significant. It is worth mentioning that In and Re were not present in the pristine materials and that they were also not detected during the in vitro release tests. This fact confirmed the absence of contamination with these elements during ALIPS9, ALIG30@10 and ALIG30@20 formulation processes and preservation.

### 3.3. Biocompatibility of ALIG30@20

**Figure 2:**
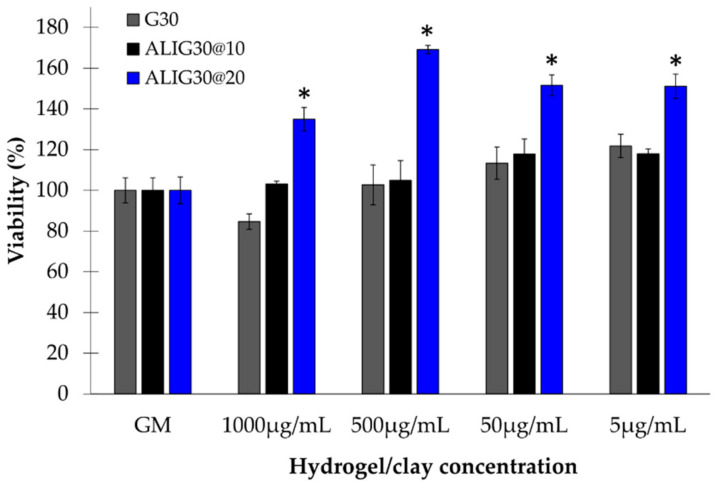
Biocompatibility tests of ALIG30@20 (blue). Viability (%) vs. hydrogel or clay concentration (% w/w). GM (growth medium) indicates the control. G30 and ALIG30@10 results (taken from García-Villén et al. [17]) were included to compare viability results of hydrogels with different concentrations. Mean values ± s.e.; *n* = 8. Significant differences, compared to GM, are marked with (*). Mann–Whitney (Wilcoxon) W tests, *p* values ≤ 0.05.

Biocompatibility results of ALIPS9@20 are reported in Figure 2. As previously mentioned, G30 and ALIG30@10 results have already been assessed by García-Villén et al. [17]. The reduction in viability produced by the pristine G30 alone at 1 mg/mL was not found in any of the hydrogels. In fact, the viability results of ALIG30@20 demonstrated, once again, that the type of formulation exerts a significant role in the results. That is, despite all tests subjected to the same amount of clay mineral in the culture, the hydrogels increased the biocompatibility. In particular, ALIG30@20 showed cellular viabilities higher than 100% at every concentration (*p* > 0.05 with respect to GM, Figure 2). In view of the experimental results and the statistical analysis, it is possible to state that ALIG30@20 exerts proliferative effects over fibroblasts at the tested concentrations. No other internal statistical differences were found between ALIG30@20 concentrations.

## 4. Discussion

### 4.1. Release of Elements and Potentially Useful Therapeutic Activities

According to the ICH Q3D(R1) guideline, no PDE limits have been established for class 4 elements [32]. The presence of Al in cosmetics is allowed according to EC 1223/2009 since it specifies that “natural hydrated aluminum silicates (Al_2_O_3_·2SiO_2_·2H_2_O) containing calcium, magnesium or iron carbonates, ferric hydroxides, quartz-sand, mica, etc. as impurities” are allowed. Aluminum has shown to be released from 1-month-old hydrogels (Table 2). The WHO has established a tolerable weekly intake of 7 mg/kg of body weight for aluminum [37]. In view of the low bioavailability of aluminum from cosmetic products (≤0.07%) [38,39,40], applications with more than 213 kg of hydrogel would be necessary to subject patients to potentially dangerous Al doses. Therefore, it is possible to guarantee that ALIPS9, ALIG30@10 and ALIG30@20 are totally safe regarding aluminum release. Additionally, some Al^3+^ “misfolds cell membrane proteins”, which gives it antibacterial activity [41].

Ca, Fe, Mn, Zn and S are not listed in this regulation [33], which means that, legally speaking, the presence of these elements does not limit the use of the present hydrogels as cosmetics from a legal point of view. Major elements such Mg, Ca, Na and K are considered as “essential” for both animals and human beings, and their presence in the pristine materials is considered totally safe and, sometimes, even favorable in certain cases. The usefulness of metals during wound healing has also been pointed out by some studies. For instance, it has been demonstrated that wound supplementation of Zn, Cu and Mg would be advisable during the healing process [42].

The amount of K in solids was higher than Na and Ca, though its release from hydrogels was remarkably lower than that of Ca and Na. This result is in agreement with the cation exchange capacity (CEC) of PS9 and G30 reported in previous studies [17], which showed calcium as one of the main exchangeable cations. Additionally, Ca is the second most abundant element in ALI. It has been reported that low concentrations of extracellular potassium may accelerate and favor fibroblast differentiation, thus forming scar tissue [43]. Low intracellular K^+^ concentrations favor interleukin-8 expression, which plays an important role in stimulating re-epithelialization, migration and proliferation of dermal cells during wound healing [26]. Therefore, a limited potassium release from both hydrogels would be beneficial during wound healing treatments. 

Sodium is the second/third element with higher in vitro release levels (Table 2) and the third/forth element in terms of abundance in the pristine materials (Table 1). Moreover, Na was one of the minor exchanged cations of PS9 and G30. This apparently contradictory result has previously been observed for other clay-based hydrogels subjected to the very same in vitro release methodology [44]. This result could be related to the hydrophilicity of the exchangeable cations of the clay, that follow the order Ca^2+^ > Na^+^ > K^+^ [45]. The higher the hydrophilicity of the element, the higher the ability of water to enter the interlayer space and the higher the exchange capacity. The very same trend has been found for Ca, Na and K release (Table 2) and CEC [17], despite this not being the same exact order of abundance in the pristine materials (Table 1). 

Mg release increased with time in ALIPS9 and ALIG30@10, whereas it reduced in ALIG30@20 (Table 2). This element has been shown to easily permeate the skin [46] and possess anti-inflammatory activity, and is thus able to treat skin disorders such as psoriasis and atopic dermatitis [47,48]. The combination of Mg and Ca has been reported to accelerate skin barrier repair, as well as skin hydration by synergic effects [49]. Moreover, apart from the beneficial effects of Mg in the skin, this element, along with Ca, is also essential for good bone and muscle health. Therefore, if any of these elements are able to reach the bloodstream during the hydrogel treatment, they could also help treat other systemic musculoskeletal disorders, such as fibromyalgia [50].

Boron compounds have been demonstrated to be beneficial for wound healing of burned skin and in diabetic wound healing processes, both in vitro and in vivo [51,52]. B has proved useful in several metabolic pathways as well as in the increase of the wound healing rate [53,54]. Release of B decreased with time in the three hydrogels until it reached undetectable levels. Consequently, if any benefit should be obtained from B, those benefits would be at its maximum in young hydrogels.

ALI composition also played an important role in the levels of elements released during the in vitro tests. In fact, the release of S can be totally ascribed to the natural spring water composition (ALI) (Table 1). The release of sulphur reduced with time in all cases (Table 2). Higher S release was reported for ALIG30@10 48 h. For ALIPS9 and ALIG30@20, the release amounts of S were very similar. Differences in ALIG30@10 and ALIG30@20 can be ascribed to the clay mineral concentration. Balneotherapy with sulphurous waters and peloids has been proven to help with several disorders and diseases [55,56]. Specifically, keratolytic, anti-inflammatory, keratoplastic and antipruritic effects have been related to S [57]. Sulphurous mineral waters may be absorbed through the skin causing vasodilation, analgesia, immune response inhibition, and keratolytic effects that reduce skin desquamation [58]. Moreover, S could potentiate angiogenesis (endothelial cell proliferation) and regulate skin immunity. Consequently, the mobility of this element would be positive, since it can ameliorate several skin disorders. In this particular case, to obtain the maximum beneficial effects from sulphur, young hydrogels should be used, when the mobility of this element is maximum. 

Mn works as a coenzyme in several biological processes, such as the transition between quiescent and proliferative phases of fibroblasts [59]. Nonetheless, Mn levels contained in healthcare formulations should be controlled due to possible toxic brain accumulation [60,61,62]. Levels of Mn were the same for pristine PS9 and G30 (while absent in ALI, Table 1). Consequently, it is possible to state that the release of this element is solely due to the clay mineral. Mn release increased with time in ALIG30@20, while it was not measurable in ALIG30@10, probably due to the lower concentration of G30 in this formulation. A study on the bioavailability of manganese from soils revealed that in acid soils, Mn bioavailability grows [63]. Previously it has been shown that G30 and PS9 hydrogels prepared with ALI water suffer from a reduction in pH values during the first 6 months [64]. This modification of the pH could be the explanation for a higher release of Mn after 1 month in ALIPS9 and ALIG30@20. In terms of safety, ALIG30@10 would be the safest formulation, since Mn release was not detectable during Franz cells study.

Zinc is a class 4 element, but it is not listed in EC 1223/2009. The ALIPS9 hydrogel showed an increase in Zn release with time, while ALIG30@20 showed stable levels (Table 2). The increase in Zn release in ALIPS9 and ALIG30@10 could also be related to pH changes in the formulation with time, although the literature results are contradictory [63]. Regarding safety and regulations, Zn did possess a defined PDE level in the Q3D(R1) [32] (13,000 μg/day for both oral and parenteral routes). Moreover, the WHO defined a provisional maximum tolerable daily intake amount of 18–60 mg/day for an adult of 60 kg. As previously mentioned, it has been reported that this element could compromise renal and hepatic functions when high doses reach the bloodstream. Nonetheless, Zn has also been demonstrated to be essential for keratinocyte and fibroblast proliferation, differentiation and survival. Its deficiency has been related to different disorders such as acquired acrodermatitis enteropathica, biotic deficiency, alopecia and delayed wound healing. Moreover, Zn concentration is usually higher in the epidermis than in the dermis [65,66]. Consequently, the mobility of Zn from the studied hydrogels is seen as a positive and potentially useful feature for wound healing. Moreover, the released amount of Zn in Franz cells can be considered safe, since it was below the WHO and PDE limits previously mentioned and they are intended to be topically administered. 

Together with Zn, Cu is a useful element in terms of wound healing [67] and its presence is allowed in cosmetics by EC 1223/2009. This element has been demonstrated to increase the expression of TGF-β1 in ex vivo skin models, thus leading to higher pro-collagen 1 and elastin production by fibroblasts [67]. Moreover, Cu has been demonstrated to enhance skin cell migration (keratinocytes and fibroblasts), which is crucial for wound healing [68,69]. ALIPS9 and ALIG30@10 were shown to favor fibroblast migration in a previous study [17], which could be related to copper release. Additionally, copper possesses an antimicrobial effect and has been proposed as an ingredient for wound dressings [70]. In fact, some clay minerals with Cu were demonstrated to be the most effective against *Escherichia coli* and *Staphylococcus aureus*. Release levels of Cu revealed that, to obtain the aforementioned effects, extemporaneous hydrogels should be used (Table 2).

Ga showed minimum mobility in both hydrogels (Table 2) and significantly reduced mobility in ALIG30@20 after 1 month. Higher release levels in ALIG30@20 versus ALIPS9 can be ascribed to a higher concentration of this element in G30 pristine material (Table 1). This element is not addressed in any of the aforementioned regulations [32,33,71,72] since it is currently considered a relatively non-toxic element for humans. Antimicrobial activity of Ga has been reported [73,74], which could be of use for the treatment of infected wounds. A biocompatible, gallium-loaded, antimicrobial, artificial dermal scaffold has been recently proposed [75]. Other biomedical uses of Ga have also been previously reported due to its low toxicity [76,77,78,79,80,81]. In view of the existing bibliography and the present results, extemporaneous ALIG30@20 hydrogels would be a proper choice to obtain antimicrobial activity.

Strontium mobility was one of the most remarkable among the trace elements, mainly because of its presence in ALI. The presence of this element in cosmetics is not considered determinant in terms of safety, maybe because symptoms of Sr overdose are not yet clear in humans. What is more, despite the in vivo studies performed in animals, no Sr limits have been established for humans (since dietary intake variations did not induced acute toxicity symptoms) [82,83]. Wound healing effects of strontium chloride hexahydrate has been evaluated in vivo. This strontium salt was shown to reduce TNF-α expression in the wound site and, therefore, reduce inflammation [84], which is of special use in chronic inflammatory disorders. The antioxidant effect is also related to Sr, according to previous studies [85] that used strontium-substituted bioglass for tissue engineering purposes. Strontium has also been included in wound dressings as a wound healing promoter [86] and has been demonstrated to exert useful systemic effects when it reaches the bloodstream [87,88,89,90]. In conclusion, the release of Sr release is desirable, ALIPS9 being the formulation providing the highest levels of this element.

### 4.2. Mobility of Elements

**Figure 3:**
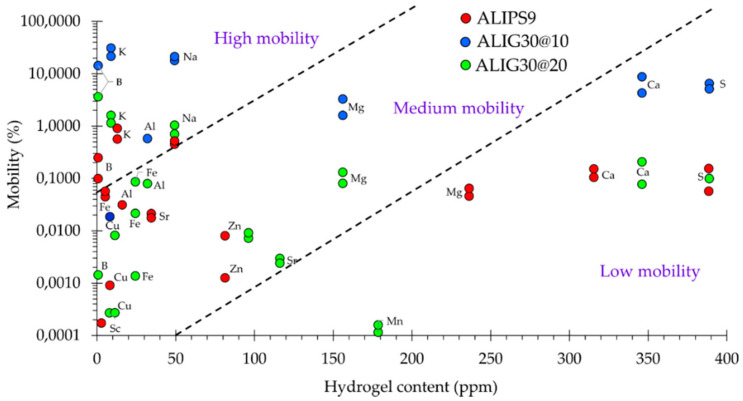
Percentage of mobility (logarithmic scale) versus total content of the element in ALIPS9, ALIG30@10 and ALIG30@20 hydrogels (ppm). “High”, “Medium” and “Low mobility” areas are hypothetical. Non-detected elements (mobility = 0%) do not appear in the logarithmic scale.

The sole presence of an element or chemical compound in a formulation does not mean that it would exert its therapeutic effect: it also needs to be released and be able to reach the active site. Moreover, the release process can be determined by different factors, one of them being its location in the formulation (clay structure or the spring water) or the age of the system [36,91]. Element mobility is a normalized parameter that allows comparisons between released levels of different elements. It can be calculated as the ratio between total concentration in the formulation and the released concentration. Mobility values of elements in ALIPS9 and ALIG30@20 hydrogels are plotted in Figure 3. In this figure, the delimited areas within the graphic were defined in a speculative manner. As can be seen from the dispersion (Figure 3), the majority of the elements showed a mobility lower than 2%.

Even if Ca, S and Mg were present in remarkable amounts in the studied formulations, their released levels were very low in proportion, thus giving rise to low element mobility. This result demonstrates that, despite the spring water having remarkable amounts of these elements, their mobility is probably limited by the presence of the solid phase. Consequently, the solid and the liquid phases of the formulations establish a very close interaction that affects the final performance of the system, something that highlights the necessity to fully characterize this kind of formulation. Another visible result is the higher mobility of elements in ALIG30@10 with respect to ALIG30@20 and ALIPS9, which also demonstrates that the type and the concentration of the clay mineral exert a remarkable influence. Elements in the “medium mobility” area (Figure 3) were located in this section since they have low mobility (<1%) together with low concentration (<150 ppm) in the final formulation. 

In view of the mobility results, K, Na, B and Al are the elements with the highest mobility. They showed relatively low amounts in the hydrogels but their mobility was clearly significantly higher with respect to the rest of the elements. We hypothesized that the high mobility of the aforementioned elements could be related to both the hydrophilicity of cations (previously mentioned in Section 4.1) and to a small/absent interaction between the pristine ingredients and, therefore, the released levels ascribed to the influence of the liquid phase (ALI) more than to the solid phase. That is, even if K, Na and B were not the main major elements in the pristine ingredients, the low interaction between K, Na and B (coming from ALI) with fibrous clay structure let these elements be relatively “free” within the system and, therefore, more prone to move. This hypothesis is confirmed by the fact that the mobility of elements in ALIG30@10 is higher than in ALIG30@20, due to the lower amount of G30 in the former. In this formulation, the reduced amount of clay mineral implies less retention of the elements and, therefore, higher mobility.

**Figure 4:**
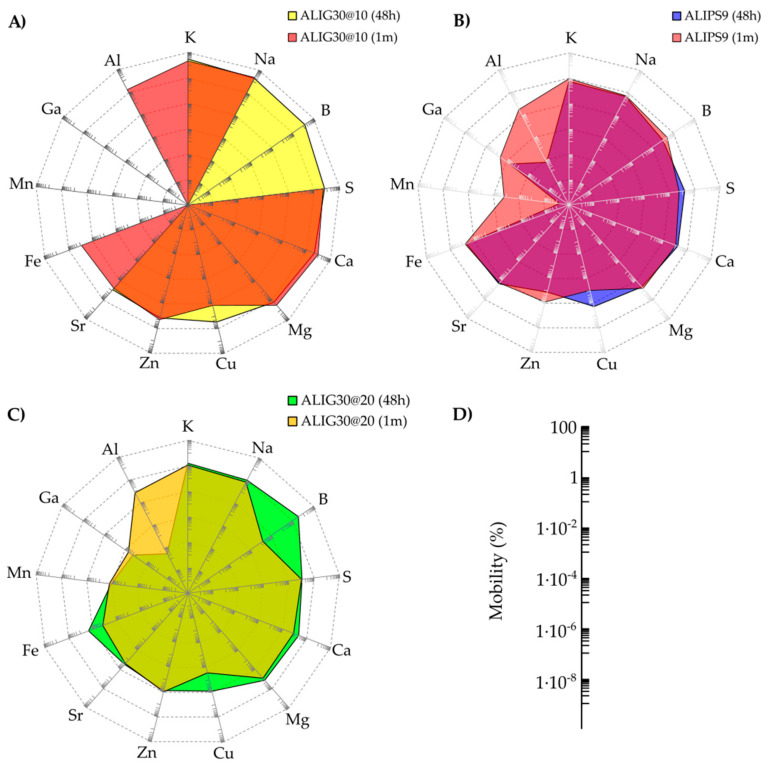
Spider diagrams of element mobility. (**A**) ALIG30@10; (**B**) ALIPS9 (**C**) ALIG30@20. For simplicity, the scale of the diagrams has been represented independently in (**D**).

Spider diagrams represent more clearly the different mobility of elements between the same hydrogels at 48 h and 1 month (Figure 4). This comparison reveals that nanoclay/spring water hydrogels are “living formulations” since their ingredients constantly interact with each other, changing the final properties of the system. The area of ALIG30@10 (48 h and 1 month) is higher than the area of ALIPS9 and ALIG30@20, which is in agreement with the previous mobility results (Figure 4). The “liveliness” of the hydrogels can be ascribed to the different elemental equilibriums established between the solid and liquid phases in the formulation (adsorption and desorption equilibriums). Upholding this hypothesis, the solid phase mainly influenced the time-mobility of Cu, Mn, Ga, Al, B, and Fe, either increasing or reducing the corresponding mobility, depending on each particular case.

**Figure A1:**
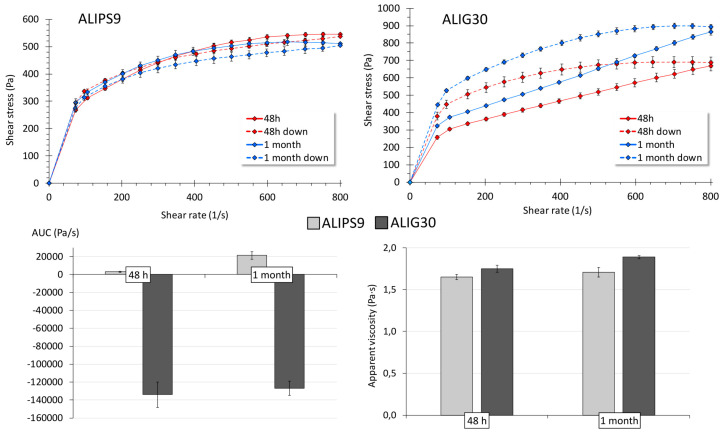
Rheological characterization of ALIPS9 and ALIG30@20 hydrogels after 48 h and 1 month. Up: flow curves (from 70 to 800 s^−1^, mean values ± s.d., *n* = 6). Down: hysteresis areas (mean values ± s.d., *n* = 6) and apparent viscosities (250 s^−1^, mean values ± s.d., *n* = 6). Positive *AUC* values indicates thixotropic behavior, while negative ones correspond to anti-thixotropic profile.

The reduction of some elements’ mobility with time (for instance B, Mg, Al, Zn, Mn, and Na) could also be explained by the stabilization of the system, and the clay better adsorbing/retaining these elements as time passes. In fact, clay minerals have been widely used for decontamination purposes due to their remarkable adsorptive properties [92,93,94,95]. Moreover, rheological changes have also been detected is these samples. A different structure of the system network could modify the mobility of certain elements and vice versa [91]. As can be seen in Appendix A (Figure A1), both ALIG30@20 and ALIPS9 suffered rheological changes within one month. Moreover, it is also possible from these results to hypothesize that the rheological performance of the system could also be influencing the element mobility. ALIPS9 and ALIG30@20, having a much more structured internal network (Figure A1), could hinder the mobility of elements that will find a more intricate path to travel towards the exterior. On the other hand, ALIG30@10 was shown to have a less structured gel network (see García-Villén et al. [64] for information on the rheology of ALIG30@10).

### 4.3. Biocompatibility of ALIG30@20

In vitro biocompatibility of clay minerals has been widely studied [15,96,97,98,99]. Some clay minerals have already been shown to have proliferating activity in cellular cultures, such as montmorillonite and halloysite [100,101]. Nonetheless, the induction of cellular proliferation by palygorskite clay mineral is a rare result [102]. This result leads us to hypothesize that if ALIG30@10 was biocompatible and able to induce fibroblast motility during in vitro wound healing [17], ALIG30@20, with proliferative activity, is also a promising formulation for wound healing treatments, especially during the proliferative stage. The different performance between these two hydrogels could be due to physicochemical differences of the systems. That is, different rheological behaviors as well as different chemical performances of both hydrogels could be the factors governing the biocompatibility results. Moreover, the present results could also be due to the combination of both physical and chemical performances of the formulations. Table A1 shows the theoretical amount of mobile element released in the fibroblast culture during MTT tests. These calculations have been made in order to correlate Franz cells results with those of MTT.

Mn has been reported as an active ingredient of spring waters with wound healing activity [29] This, together with the Mn released results in ALIG30@10 and ALIG30@20 (Table 2), leads us to propose manganese as one of the possible factors explaining the proliferative effect of ALIG30@20 versus ALIG30@10 (Figure 2).

Calcium and zinc have been demonstrated to actively participate in cellular growth, in particular the Zn:Ca ratio, which was demonstrated to increase Zn:Ca during cell proliferation and the decline Zn:Ca during the remodeling phase [20,21,103]. This is due to a redistribution of calcium within dermal cells during the wound healing cascade [104], which is dependent on certain trace elements such as zinc. In fact, extracellular calcium has been shown to stimulate DNA synthesis in cultured fibroblasts in the presence of Zn [105]. This has been mainly ascribed to the cofactor role of Zn in different enzymes involved in fibroblast growth. Moreover, Zn also plays an important role as a structural component of essential proteins. Some in vitro studies demonstrated that, even if proper growth factors and nutrients are present in the fibroblast culture medium, deficiencies of Zn translate to insufficient intracellular calcium and, ultimately, to impaired fibroblast proliferation [106,107]. From the release values of these elements, the Zn:Ca ratio of ALIG30@10 was 0.00465 and 0.01060 for ALIG30@20 (obtained from Table A1), which could be a significant factor inducing the proliferation of fibroblasts in ALIG30@20. It is also worth pointing out the fact that G30 showed a remarkable amount of Zn, thus being the ingredient providing this element. On the other hand, the major amount of Ca is provided by ALI. Any of the formulation ingredients on their own have been shown to induce cellular proliferation (see MTT results in García-Villén et al. [17] and Figure 2). This indicates that both ALI and G30, properly combined in a certain concentration, are necessary to induce fibroblast proliferation. Consequently, the proliferative effect is ascribed to the formulation itself, proving once again the major importance of formulative studies. By the same token, the Ca:Mg ratio also changes along the wound healing cascade. In fact, an increase in Mg levels is observed to favor cellular migration. Grzesiak and Pierschbacher stated that the Mg:Ca ratio was close to 1 during the migratory phase, and it reversed during the rest of the process [108]. ALIPS9 and ALIG30@10 hydrogels (aged for 1 month) showed Mg:Ca ratios (Table 2) closest to 1, which is in agreement with the induction of fibroblast migration already demonstrated for these formulations [17]. Nonetheless, the ALIG30@20 Mg:Ca ratio was significantly distant from this value, which happens during the proliferative phase.

The present results ultimately lead us to think that, apart from the amount of elements released from each hydrogel, their ratio and specific identity highly influence the final therapeutic performance of the formulation. Notwithstanding the fact that further studies are needed, it is noteworthy that the present formulations have the potential to be combined and administered at different times of the wound treatment by virtue of their chemical performance

## 5. Conclusions

The present study deals with the in vitro release and mobility of potentially bioactive elements present in semisolid gel-like formulations obtained by mixing sepiolite and palygorskite with a natural spring water. Hydrogels were subjected to in vitro Franz cell tests and the elements released were analyzed by inductively coupled plasma techniques. Then, the element release and mobility were compared with in vitro biocompatibility tests of the very same formulation. The results demonstrated that, unlike other formulations, the potential therapeutic activity of nanoclay/spring water hydrogels should be studied in depth and characterized.

Clay/spring water hydrogels are “living formulations” since their ingredients constantly interact with each other, changing the properties of the system. For instance, the presence of an element in high concentration does not mean it would be released in high amounts. Moreover, the high release of bioactive elements is not a *sine qua non* to obtain maximum therapeutic effect. In fact, the ALIG30@20 hydrogel, with lower elemental mobility, not only proved to be biocompatible, but to exert potential proliferative effects over fibroblast cultures. According to the present in vitro release studies, it is possible to state that the ratios of the elements released play a significant role in the final therapeutic activity of the formulation. Moreover, the importance of formulative studies is again highlighted, since it is the optimal combination of the correct ingredients that makes a formulation effective.

As a general conclusion, the present study demonstrates that synergistic effects can be achieved from the formulation of the liquid phase in a semisolid system, in which elemental composition of the solid phase and structure of the system will determine elements’ mobility and, ultimately, the therapeutic effects.

## Figures and Tables

**Table 1:** Elemental composition of pristine samples (PS9, G30 and ALI) determined by ICP-OES and ICP-MS. “ND” stands for “Not Detected”. For a better understanding, comments about each element are included within the table. Levels of elements marked with * were obtained from [36].

Element	PS9 (ppm)	G30 (ppm)	ALI (ppb)	Comments
Al	15.9	31.9	37	Class 4 Q3D(R1); Allowed in EC 1223/2009
B	0.3	0.3	395	Class 4 in Q3D(R1); Not listed in EC 1223/2009
Ca	2.8	33.3	312,700	Class 4 in Q3D(R1); Not listed as element in EC 1223/2009
Fe	5.2	24.2	58	
K	6.0	1.9	6836	
Mg	122.0	41.8	114,267	
Na	0.1	0.1	49,150	
S	0.1	0.4	388,367	Not listed as element in EC 1223/2009
Mn	177.0	178.5	ND	Class 4 in Q3D(R1); Not listed in EC 1223/2009
W	0.9	0.4	ND	
Zn	81.2	96.1	3.3	Class 4 in Q3D(R1); Not listed as element in EC 1223/2009
Cu *	8.1	11.3	2.5	Class 3 in Q3D(R1); Allowed in EC 1223/2009
Ag *	0.04	0.2	0.1	Class 2B in Q3D(R1); Allowed in EC 1223/2009.
Au *	ND	ND	ND	
Sc	2.7	7.9	1.8	Not listed in EC 1223/2009
Ti	689.6	1820.5	0.2	Not listed as element in EC 1223/2009
Ga	8.2	16.1	0.8	Not listed in EC 1223/2009
Ge	3.2	0.8	0.1	
Tb	43.2	17.9	7.2	
Sr	24.4	106.0	10,049	Not listed as element in EC 1223/2009
Y	6.2	39.9	0.04	Not listed in EC 1223/2009
Nb	3.8	6.1	0.002	
In	ND	0.002	ND	
La	7.7	36.3	ND	
Ce	17.1	48.9	ND	
Pr	2.0	7.5	0.003	
Sm	1.7	5.5	0.001	
Eu	0.2	1.2	0.001	
Gd	1.5	5.6	0.003	
Dy	1.2	4.7	0.002	
Ho	0.2	1.0	0.002	
Er	0.6	2.9	0.002	
Tm	0.1	0.4	0.002	
Yb	0.5	2.4	ND	
Lu	0.1	0.4	0.002	
Hf	44.7	13.9	2.3	
Re	ND	ND	0.01	
Bi	0.1	ND	ND	Not listed as element in EC 1223/2009
Th	4.6	5.6	0.1	Not listed in EC 1223/2009

**Table 2:** Mobility of elements after Franz diffusion cell tests. Major elements are expressed in mg/100 g of hydrogel, while the rest of the elements are expressed as μg/100 g of hydrogel. Mean values ± s.e. (*n* = 6). “ND” stands for “Not Detected”. Release levels of elements marked with * were obtained from [36].

Concentration Units	Element	ALIPS9		ALIG30@10		ALIG30@20	
		48 h	1 m	48 h	1 m	48h	1 m
mg/100 g	Ca	11.7 ± 2.91	8.1 ± 1.30	14.9 ± 1.758	30.4 ± 7.379	17.5 ± 3.51	7.0 ± 1.25
	K	1.8 ± 0.843	2.8 ± 0.628	2.7 ± 1.183	1.9 ± 0.491	3.4 ± 1.004	2.6 ± 1.09
	Mg	2.7 ± 0.48	3.7 ± 0.52	2.5 ± 0.237	5.2 ± 1.433	4.9 ± 0.23	3.0 ± 0.48
	Na	5.4 ± 1.40	6.3 ± 1.65	8.8 ± 2.727	10.5 ± 3.185	12.4 ± 1.136	6.43 ± 0.469
	S	14.8 ± 3.80	5.5 ± 2.81	23.3 ± 2.063	11.7 ± 2.162	10.4 ± 2.34	6.7 ± 2.29
	B	0.2 ± 0.016	0.04 ± 0.020	0.1 ± 0.021	ND	0.3 ± 0.062	ND
	Fe	0.06 ± 0.036	0.07 ± 0.028	ND	0.02 ± 0.018	0.1 ± 0.054	0.01 ± 0.009
	Al	ND	0.1 ± 0.056	ND	0.58 ± 0.452	ND	0.67 ± 0.100
μg/100 g	Mn	ND	0.7 ± 0.39	ND	ND	4.9 ± 2.64	7.4 ± 3.69
	W	ND	ND	ND	ND	ND	ND
	Zn	25.9 ± 16.07	165.9 ± 68.51	132.1 ± 38.17	181.4 ± 99.18	164.8 ± 53.09	175.1 ± 80.91
	Cu*	10.8 ± 3.29	3.6 ± 2.17	32.6 ± 11.59	1.5 ± 1.01	20.6 ± 3.725	0.9 ± 0.61
	Ag *, Au *, Sc, Ti, Ge, Tb	ND	ND	ND	ND	ND	ND
	Ga	ND	0.08 ± 0.050	ND	ND	0.2 ± 0.019	0.04 ± 0.001
	Sr	176.5 ± 15.89	148.7 ± 20.37	90.4 ± 18.67	65.9 ± 10.39	82.8 ± 15.64	68.5 ± 7.93
	Y, Nb, In, La, Ce, Pr, Sm, Eu, Gd, Dy, Ho, Er, Tm, Yb, Lu, Hf, Re, Bi, Th	ND	ND	ND	ND	ND	ND

## Appendix A

**Table A1:** Amount of mobile elements released from the corresponding hydrogels into fibroblast cultures (MTT well-plate). Results were calculated from Franz experiments at 1 month and expressed in nanograms. NM stands for “Not Released”.

Hydrogel	Element (ng)	Amount of Hydrogel in Cell Wells During MTT Test			
		1000 μg/mL	500 μg/mL	50 μg/mL	5 μg/mL
ALIPS9	Al	2.25	1.13	0.11	0.01
	B	0.79	0.39	0.04	0.004
	Ca	161.5	80.7	8.08	0.81
	K	55.3	27.6	2.76	0.28
	Mg	73.8	36.9	3.69	0.37
	Na	125.7	62.8	6.28	0.63
	S	109.4	54.7	5.47	0.55
	Fe	1.46	0.73	0.07	0.01
	Sr	2.97	1.49	0.15	0.01
	Zn	3.32	1.66	0.17	0.02
	Mn	0.01	0.01	0.0007	0.0001
	Cu	0.07	0.04	0.0036	0.0004
ALIG30@10	Al	11.6	5.78	0.58	0.06
	B	NR	NR	NR	NR
	Ca	608.3	304.1	30.41	3.04
	K	38.3	19.1	1.91	0.19
	Mg	103.1	51.6	5.16	0.52
	Na	210.7	105.3	10.53	1.05
	S	234.0	117.0	11.70	1.17
	Fe	0.42	0.21	0.02	0.002
	Sr	1.32	0.66	0.07	0.01
	Zn	2.83	1.41	0.14	0.01
	Mn	NR	NR	NR	NR
	Cu	0.036	0.018	0.002	0.0002
ALIG30@20	Al	6.72	3.36	0.34	0.03
	B	0.001	5·10^4^	5·10^-5^	5·10^-6^
	Ca	165.2	82.6	8.26	0.83
	K	25.9	12.9	1.29	0.13
	Mg	29.7	14.8	1.48	0.15
	Na	84.8	42.4	4.24	0.42
	S	79.4	39.7	3.97	0.4
	Fe	0.09	0.04	4.4·10^-3^	4.4·10^-4^
	Sr	0.69	0.34	0.03	0.003
	Zn	1.75	0.88	0.09	0.01
	Mn	0.07	0.04	0.0004	3.7·10^-4^
	Cu	0.01	0.005	4.6·10^-4^	4.6·10^-5^
